# A case of Kounis syndrome after a hornet sting and literature review

**DOI:** 10.1186/1756-0500-7-867

**Published:** 2014-12-03

**Authors:** Dissanayake Mudiyanselage Priyantha Udaya Kumara Ralapanawa, Senanayake Abeysinghe Mudiyanselage Kularatne

**Affiliations:** Department of Medicine, University of Peradeniya, Peradeniya, Sri Lanka

**Keywords:** Kounis syndrome, Acute coronary syndrome, Vespa affinis, Sri Lanka

## Abstract

**Background:**

Acute coronary syndrome after hymenoptera stings or exposure to environment toxins is referred to as the Kounis syndrome or allergic myocardial ischaemia with or without infarction. We report a case of hornet (Vespa affinis) sting causing Kounis syndrome in Sri Lanka and present a review of literature.

**Case presentation:**

A 60-year -old female with diabetes mellitus and known allergy to bee venom was stung by a hornet on the right hand. Within 30 minutes she developed hypotension and wide spread T wave inversion in the 12 leads ECG that remained unchanged about 5 hours and reversed back to normal.

**Conclusion:**

Hymenoptera stings can induce acute coronary syndrome either by direct effect of venom constituents on the coronary endothelium or through inflammatory mediators induced allergic reaction on coronary vasculature. Early recognition of Kounis syndrome is needed in hornet stings to implement necessary treatments.

## Background

**Figure 1:**
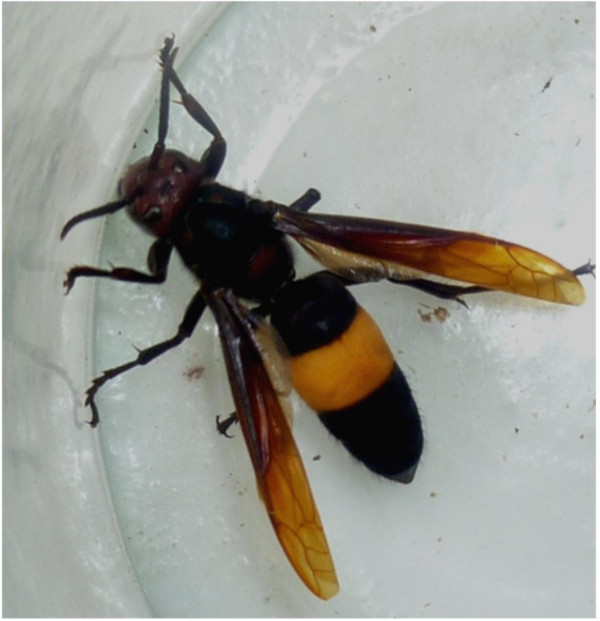
**Hornet, Vespa affinis.**

The hymenoptera including the stinging hornets and bees are widely distributed in Sri Lanka and their stinging is a common environmental hazard causing significant unaccountable morbidity and mortality. The hornet in Sri Lanka is Vespa affinis or Debara in Sinhala of the genus Vespa, commonly build their nests in peridomestic enviorenment [1]. The length of a hornet ranges from 2 to 3 cm and its mid body has a yellow band separating its brownish red front from its black hind part (Figure 1).

The hornet can inflict multiple stings because the stinger has no barbs unlike bees and does not get detached after stinging [1]. The venom of a hornet contains a mixture of histamine releasing factors, serotonin, prostaglandins, leukotrients, thromboxane, haemolysins, vasodilators, vasospastic amines and phospholipase A [1].

Hornets protect their nests and any disturbance provokes them to attack people in the vicinity causing multiple sting injuries. The complications of stinging sometime unpredictable and may result in deaths [1]. The histamine releasing action of the venom during the first contact with a victim is the most common cause of pathophysiology following hornet stings and other reported manifestations were myocardial infarction, multiple organ failure, myasthenia gravis, mastocytosis and reversible optic neuropathy [1].

## Case presentation

**Figure 2:**
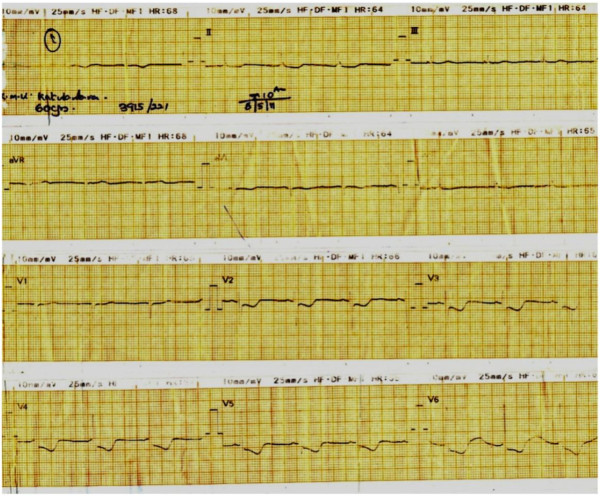
**Electrocardiogram taken just after admission.**

**Figure 3:**
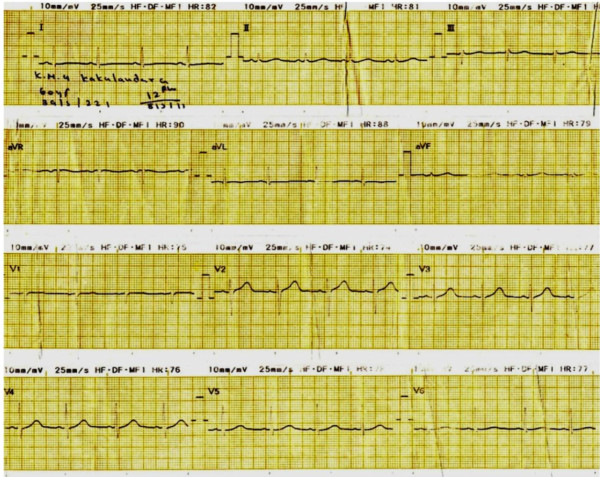
**Electrocardiogram taken five hours after admission.**

A 60-year-old female with diabetes mellitus and known allergy to bee venom was stung on the right hand by a hornet while working in the garden. Within few minutes, she felt unwell, light headed and strange feeling. In about 20 minutes, she became acutely breathless and experienced mild central chest pain and was brought to a hospital immediately. When she arrived at the Emergency Treatment Unit (ETU) in about 30 minutes after the sting, she was conscious but restless and had blood pressure of 74/50 mmHg. She had a circumscribed punctated sting mark with mild local swelling of dorsum of hand. There were no other sting marks or urticaria present elsewhere in the body. She was tachypnoic and had bilateral polyphonic rhonchi. Twelve lead ECG was taken and it showed widespread ST segments depressions and inverted T waves (Figure 2). Her peripheral an oxygen saturation (SpO_2_) was 94% and random blood sugar was 134 mg/dl. Anaphylactic shock was the diagnosis and 0.5 ml (1:1000) adrenalin im, Hydrocortisone 200 mg iv and Chlorpheniramine 10 mg iv were given immediately. She felt better and improved over next 20 minutes and her blood pressure picked up to 106/70 mmHg. Symptomatically she became better with reduced chest tightness, chest pain and breathlessness. However, the repeat ECG taken 30 minutes later showed the same changes as first ECG. She was kept under observation in ETU and she didn’t develop any new symptoms. Blood pressure remained stable. The ECGs taken three and five hours after the admission (Figure 3) were normal with disappearance of ST and T wave inversion shown in the first and second ECGs. By this time patient felt she is back to normal health and her blood pressure was 110/80 mmHg. The Troponin T level was done 12 hours after stinging and was within normal range. She was given her usual antidiabetic medications and aspirin. Medications indicated for acute coronary syndrome such as heparin, nitrates and statins were not given as ECG changes were transient and he felt free of chest pain. She was discharge from the hospital after 48 hours and was followed up in the out patients clinic. Two months later she had 2D echocardiogram and exercises ECG both were normal. Coronary angiogram was suggested but she did not give consent for it.

## Discussion

Acute coronary syndrome after hymenoptera stings and other environment exposures are referred to as the Kounis syndrome or allergic myocardial ischaemia and infarction [2, 3]. The events of our patient would fit well into acute coronary syndrome associated with allergic reaction after a hornet sting. The patient’s symptoms after the hornet sting are attributable either to anaphylaxis or to acute myocardial ischemia or to both [4, 5]. Hymenoptera stings can induce acute coronary syndromes by different pathogenic mechanisms including direct action of the venom constituents on the coronary endothelium or allergic reaction with mediators acting on the coronary vasculature.

The pathophysiology of Kounis syndrome is known to be due to inflammatory mediators released from mast cell activation induced by allergic or hypersensitivity reaction. The reaction to hymenoptera sting can be prolonged or severe in atopic individuals and in some cases anaphylaxis may ensue, with urticaria, circulatory collapse and bronchospasm. This is a results of sequence of events including the release of serotonin and histamine and the formation of leukotrienes [5], some of which are potent coronary vasoconstrictors in various animal species [6, 7]. Many pharmacologically active constituents of wasp venom have been isolated including histamine, serotonin, dopamine, noradrenalin and a bradykinin like substances which may itself induce histamine release [8]. Endogenous secretion of adrenaline and noradrenaline is stimulated by histamine and serotonin [9]. All these substances can provoke myocardial ischemia either through profound hypotension or by increasing myocardial oxygen demand through direct ionotropic or chronotropic effect in the presence of a compromised myocardial blood supply. Platelet aggregation is induced by serotonin and adrenaline. Adrenaline also accelerates thrombus formation in animals and in man possibly by increased factor v activity [10, 11] and shown in animals to release a thromboplastin-like substance from the walls of blood vessels. It causes both coronary vasodilatation and increased myocardial oxygen demand by direct ionotropic and chronotropic effect. Adrenaline has been used historically as a provocation test for angina pectoris and often used in the treatment of anaphylactic shock [11].

In the course of anaphylactic reaction, complements are activated with anaphylatoxin generation. The specific receptors for them are present on surface of cardiac mast cells [12]. The final step of this processes is mast cells degranulation, resulting in histamine, tryptase and chymase release, as well as in prostaglandin and leukotriene synthesis. The histamine concentration is elevated in arterial walls containing atheromatic changes [12]. Also proteases (Tryptase, Chymase and Cathepsin-D) released from stimulated mast cells activate metalloproteinases (MMP: MMP-1, MMP-3 and MMP-9) which degrade connective tissue covering the atheromatous plaque. The plaque becomes vulnerable i.e. more prone to rupture [12]. Patrizia Bonadonna et al. [13] show that systemic reactions to Hymenoptera stings with variable severity occur in up to 5% of the adult population in Europe and the United State. Also pointed out subjects with mastocytosis (whom basal serum trypsin level high) might experience more severe reactions after Hymenoptera stings [13].

There are three variants of Kounis syndrome [14]. Type 1 variant includes patients with normal coronary arteries without predisposing factors for coronary artery disease, in whom the acute allergic insult induces either coronary artery spasm with normal cardiac enzymes and troponins or coronary artery spasm progressing to acute myocardial infarction with raised cardiac enzymes and troponins. This variant might represent a manifestation of endothelial dysfunction or micro vascular angina. Type II variant includes patients with quiescent pre-existing atheromatous disease in whom acute allergic episode can induce plaque erosion or rupture manifesting as an acute myocardial infarction [15, 16]. Several reports have shown that type I variant of Kounis syndrome has better prognosis than type II variant. However, in both types prognosis depends on the magnitude of the initial allergic response, the patient’s sensitivity, co-morbidity, the site of antibody antigen reaction, the allergen concentration and the route of allergen entrance [16]. A type III variant has been described as coincidence of hypersensitivity reactions following implantation of drug-eluting stents, causing stent thrombosis [14]. Our patient is 60 years old and had type 2 diabetes mellitus as predisposing factors for coronary artery disease. She had no previous history of angina and her 2D Ehocardiogram and exercise ECG were normal. But without coronary angiogram it is difficult to say which type of Kounis syndrome she actually had.

The chest pain of the patient occurred within minutes after the sting but before adrenalin was given. Also her ECG changes appeared before giving adrenalin. These evidence supports occurrence of acute coronary event as a direct result of the hornet sting. On the other hand hypotension caused by anaphylaxis can leads to myocardial hypo perfusion and acute ischaemia. Even though, patient recovered from anaphylactic shock within few minutes of emergency treatment, it was taken several hours for the reversal of ECG changes. It may be possibly due to prolonged effect of hornet venom and released mediators on myocardium and coronary vasculature.

With regards to the therapeutic approach of coronary spasms following an allergic reaction, the medications should include vasodilators, such as nitrates, and calcium channel inhibitors, which are in any case the treatment of choice in every case of coronary spasm. In contrast, the role of corticosteroids and antihistamines, apart from their clear usefulness in the treatment of systemic manifestations of the allergy, has not been fully determined. In other words, it is not known to what extent these, and other pharmaceutical agents have a stabilizing action of the membrane of mast cells or restrict the action of mediators of the allergy, or have a role in the treatment of acute coronary events that are caused by allergic reactions [15]. Drugs including mediator antagonists, inhibitors of mediator biosynthesis, leukotriene antagonists, mediator receptor blockers such as sodium nedocromil, sodium cromoglycate, ketotifen, Idoxamide, humanized IgG1 monoclonal antibodies and others which interfere with mast cell stabilization and prevent the release of mast cell contents, could emerge as novel therapeutic modalities capable of preventing acute coronary syndrome [14].

## Conclusion

Although, allergic episodes are common in everyday practice, early recognition of Kounis syndrome in hornet sting is important to provide needed treatments. In future, further studies to understand the mechanisms of allergy causing acute coronary syndromes are needed as this will pave way to effective therapeutic interventions.

## Consent

Written informed consent was obtained from the patient for publication of this case report and accompanying images. A copy of the written consent is available for review by the editor-in-chief of this journal.

## Authors’ information

DMPUKR (MBBS, MD, MRCP, FRCP) is a Consultant Physician and Senior Lecturer in medicine.

SAMK (MBBS, MD, MRCP, FCCP, FRCP) is Senior Professor & Consultant physician.

## Abbreviations

ECG: Electrocardiogram
ETU: Emergency treatment unit
2D: Two dimensional
IgG1: Imunoglobulin 1.
